# A radiotherapeutic paradox: ellagic acid sensitizes tumors while attenuating radiation-induced myocardial injury

**DOI:** 10.3389/fonc.2026.1652278

**Published:** 2026-01-28

**Authors:** Dandan Li, Siqin Xie, Sisi Yang, Chenghao Zhu, Haomiao Lan, Ke Zhu, Ziyang Zhu, Kun Wang, Fuqiang Shao

**Affiliations:** 1 Department of Nuclear Medicine, The First People’s Hospital of Zigong, Zigong, Sichuan, China; 2 Department of Ultrasonography, Wuhan Children’s Hospital (Wuhan Maternal and Child Health Care Hospital), Tongji Medical College, Huazhong University of Science and Technology, Wuhan, China; 3 Department of Thyroid & Breast Surgery, Zigong First People’s Hospital, Zigong Academy of Medical Sciences, Zigong, China; 4 Medical Department, Wuhan City College, Wuhan, China; 5 Department of Nuclear Medicine, Sichuan Provincial People’s Hospital, University of Electronic Science and Technology of China, Chengdu, China; 6 Department of Nuclear Medicine, Shanghai East Hospital, School of Medicine, Tongji University, Shanghai, China

**Keywords:** ellagic acid, radiation-induced myocardial injury, radiosensitizing, radiotherapy, triple-negative breast cancer

## Abstract

**Background:**

Triple-negative breast cancer (TNBC) is a highly aggressive subtype of breast cancer lacking effective targeted therapies. Radiotherapy remains a mainstay for locoregional control, but its efficacy is limited by intrinsic tumor radioresistance and the risk of radiation-induced myocardial injury (RIMI), particularly during chest irradiation. This study aimed to investigate whether ellagic acid, a naturally occurring polyphenol, could simultaneously enhance radiosensitivity in TNBC while protecting cardiac tissue from radiation-induced damage.

**Methods:**

We employed both *in vitro* and *in vivo* TNBC models to assess the effects of ellagic acid on cell cycle progression, DNA damage response, oxidative stress, and cardiac injury. Immunofluorescence, histological staining, and echocardiography were used to evaluate tissue-level outcomes following fractionated radiotherapy, with or without EA treatment.

**Results:**

Ellagic acid effectively induced G1-phase cell cycle arrest and suppressed CDK4, thereby reducing S-phase entry and enhancing radiation-induced apoptosis in TNBC cells. *In vivo*, ellagic acid significantly enhanced tumor growth inhibition when combined with radiotherapy. Importantly, ellagic acid also mitigated RIMI by reducing cardiomyocyte apoptosis, preserving myocardial structure, and maintaining left ventricular systolic function. No evidence of systemic toxicity was observed.

**Conclusion:**

Ellagic acid exhibits a unique dual function in TNBC radiotherapy: it sensitizes tumors by targeting the CARM1-CDK4 axis while protecting the heart through potent antioxidant activity. These findings position ellagic acid as a promising adjuvant candidate to enhance therapeutic efficacy and safety in TNBC radiotherapy.

## 1 Introduction

Triple-negative breast cancer (TNBC), accounting for approximately 15–20% of all breast cancer cases, is characterized by the absence of estrogen receptor, progesterone receptor, and human epidermal growth factor receptor 2 expression (1–3). Due to the lack of actionable molecular targets, patients with TNBC are largely reliant on chemotherapy and radiotherapy for locoregional disease control (4). Radiotherapy remains a cornerstone in the management of TNBC, capable of significantly reducing local recurrence and improving overall survival (4). However, irradiation of the chest wall or breast inevitably exposes the heart to ionizing radiation, placing patients at risk for radiation-induced myocardial injury (RIMI) (5).

Although adult cardiomyocytes exhibit low proliferative activity and are considered less radiosensitive than cancer cells, their extremely limited regenerative capacity renders the heart vulnerable to even minor radiation-induced insults (6). Clinically, RIMI can manifest as progressive myocardial fibrosis, contractile dysfunction, arrhythmias, or even heart failure, which may develop days to months after treatment (7). The cardiotoxicity of radiotherapy is therefore a growing concern, particularly in TNBC patients who may receive aggressive thoracic irradiation (8).

The primary antitumor mechanism of radiotherapy is the induction of DNA double-strand breaks, which trigger irreparable genomic damage and apoptosis (9). However, rapidly proliferating tumor cells, particularly those in S phase, possess robust DNA repair capacity via high-fidelity mechanisms such as homologous recombination (10). Notably, preclinical studies have demonstrated that pharmacological blockade of the G1/S transition using CDK4/6 inhibitors not only impedes tumor growth but also enhances radiosensitivity, highlighting the potential of cell cycle modulation as a radiosensitization strategy (11).

Ellagic acid is a natural polyphenolic compound abundant in berries, pomegranates, and nuts (12, 13). It possesses two key properties that make it an attractive candidate in radiotherapy contexts: Potent antioxidant activity – The multiple hydroxyl groups on its aromatic rings allow efficient electron donation, enabling effective scavenging of radiation-induced reactive oxygen species (ROS), thus mitigating oxidative damage to normal tissues (14); Cell cycle regulatory activity – ellagic acid has been shown to inhibit the histone arginine methyltransferase CARM1, specifically blocking H3R17 dimethylation, which suppresses CDK4 transcription (15–17). Remarkably, ellagic acid exhibits stronger inhibitory effects on the CARM1–CDK4 axis than selective small-molecule probes such as TP-064, resulting in robust G1 arrest and prevention of S-phase entry (15).

Based on these features, we hypothesized that ellagic acid may exert a dual radiomodulatory effect: (1) enhancing radiosensitivity in tumor cells by preventing entry into the DNA-repair–competent S phase, and (2) protecting cardiomyocytes from radiation-induced oxidative injury through ROS scavenging. Despite the therapeutic promise of this dual mechanism, no prior studies have evaluated ellagic acid as both a radiosensitizer and a cardioprotective agent.

In this study, we comprehensively investigated the dual-function potential of ellagic acid using *in vitro* assays and a murine 4T1 TNBC model. Specifically, we examined its antioxidant capacity, cell cycle effects, tumor response to combined ellagic acid and radiotherapy treatment, and the extent of radiotherapy-induced cardiac injury. Our findings reveal a paradoxical yet advantageous therapeutic profile—ellagic acid sensitizes tumors to radiation while protecting the heart—supporting its translational potential in TNBC radiotherapy.

## 2 Result

### 2.1 Ellagic acid exhibits dual roles in antioxidation and tumor cell cycle regulation

**Figure 1:**
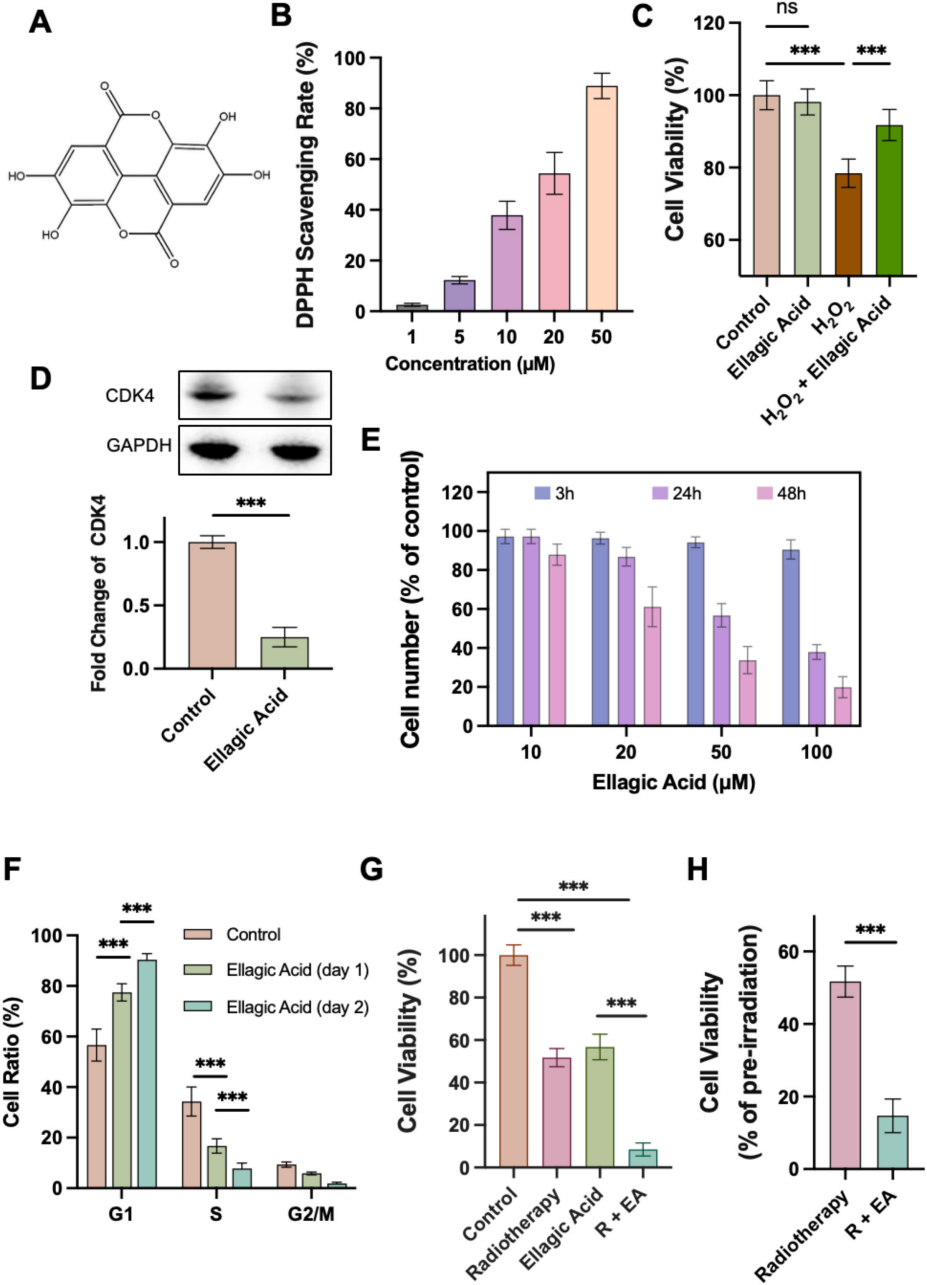
Characterization of ellagic acid and its biological effects on oxidative stress protection and tumor cell cycle regulation. **(A)** Chemical structure of ellagic acid. **(B)** DPPH radical scavenging assay of ellagic acid at indicated concentrations. **(C)** Cell viability of H9C2 cardiomyocytes under H2O2-induced oxidative stress with or without ellagic acid. **(D)** CDK4 expression levels in 4T1 cells after ellagic acid treatment. **(E)** Cell number of 4T1 cells after ellagic acid treatment for 3 h, 24 h, and 48 h. **(F)** Cell cycle distribution of 4T1 cells after ellagic acid treatment for 1 or 2 days. **(G)** Cell viability of 4T1 cells treated with radiotherapy, ellagic acid, or their combination. **(H)** Cell viability (as percentage of pre-irradiation) after radiotherapy with or without ellagic acid pretreatment. ***P < 0.001; ns, not significant. n = 5 biological replicates.

**Figure 1** illustrates the biochemical characteristics of ellagic acid and its effects at the cellular level. First, we presented the chemical structure of ellagic acid (**Figure 1A**). Ellagic acid contains multiple phenolic hydroxyl groups, which endow it with strong electron-donating capacity, enabling it to effectively scavenge free radicals and exhibit potent antioxidant activity (18, 19). To validate its antioxidant capacity, we conducted a DPPH radical scavenging assay. In this assay, the stable free radical DPPH exhibits a characteristic absorbance at 517 nm, which decreases upon reaction with hydrogen-donating antioxidants (20, 21). Ellagic acid induced a clear, dose-dependent reduction in absorbance, confirming its effective free radical scavenging ability (**Figure 1B**).

To further evaluate its protective role under oxidative stress, H9C2 cardiomyocytes were treated with hydrogen peroxide (H_2_O_2_) to induce oxidative injury, followed by a 3-hour incubation with ellagic acid. The results showed that ellagic acid significantly improved cell viability under oxidative conditions (**Figure 1C**). Ionizing radiation induces cellular damage through both direct DNA double-strand breaks and indirect mechanisms, most notably the generation of ROS such as superoxide anions, hydroxyl radicals, and H_2_O_2_ (22). Given the limited regenerative capacity of cardiomyocytes, even moderate oxidative injury may lead to progressive structural and functional deterioration (23). By scavenging free radicals and restoring redox balance, ellagic acid offers a biologically plausible and clinically relevant strategy to protect the heart from radiation-induced oxidative stress.

Given that ellagic acid is an effective inhibitor of CARM1/CDK4 mediated cell proliferation (15–17), we next examined its impact on CDK4 expression in 4T1 tumor cells. As shown in **Figure 1D**, ellagic acid treatment markedly decreased CDK4 protein levels in 4T1 cells. Since CDK4 plays a key role in the G1/S phase transition of the cell cycle, this downregulation suggests that ellagic acid suppresses tumor proliferation by blocking cell cycle progression (24).

To further confirm the antiproliferative effect of ellagic acid, we quantified 4T1 cell numbers after treatment. The results revealed a significant, dose- and time-dependent reduction in cell numbers (**Figure 1E**). Cell cycle analysis showed that ellagic acid treatment led to a marked increase in the proportion of cells in the G1 phase, which further increased over time, confirming that ellagic acid induces G1 phase arrest and inhibits G1/S transition (**Figure 1F**).

To investigate whether ellagic acid could sensitize tumor cells to radiotherapy, we performed a combinatorial treatment in which 4T1 cells were pretreated with ellagic acid for 24 hours, followed by Co60 γ-irradiation. Cell viability was then assessed using the CCK8 assay. As shown in **Figure 1G**, both radiotherapy alone and ellagic acid alone significantly reduced cell viability compared to the control group, while the combination treatment (radiotherapy + ellagic acid, R+EA) further enhanced this reduction. **Figure 1H** presents the post-irradiation viability relative to pre-irradiation levels. The R+EA group exhibited a much greater decline in viability than the radiotherapy-only group, indicating that ellagic acid effectively enhances tumor cell sensitivity to radiation. Furthermore, we also evaluated the protective effect of ellagic acid (EA) on cardiomyocytes under radiation therapy. After subjecting cardiomyocytes to Co60 γ-irradiation, EA was immediately administered, followed by ROS fluorescence staining two hours later. The results showed that EA treatment significantly reduced the ROS fluorescence signal in H9C2 cardiomyocytes (**Supplementary Figure S1**).

In summary, these findings suggest that ellagic acid possesses dual functional properties: it protects cardiomyocytes from radiation-induced oxidative damage via its antioxidant capacity, and concurrently, it inhibits tumor cell proliferation by inducing G1 phase arrest, thereby enhancing radiosensitivity. Notably, because cardiomyocytes are terminally differentiated and do not actively proliferate, ellagic acid’s cell cycle–blocking effects have minimal impact on cardiac tissue (25). This tissue-specific distinction enables ellagic acid to sensitize proliferating tumor cells to radiation while sparing the heart, further supporting its potential as a safe and effective adjuvant in radiotherapy.

### 2.2 Ellagic acid enhances radiotherapy-induced tumor suppression and apoptosis *in vivo*

To further validate the ability of ellagic acid to enhance radiotherapy efficacy *in vivo*, we established a subcutaneous 4T1 TNBC xenograft model in mice. Starting from day 1 post-implantation, ellagic acid was administered daily via intraperitoneal injection. Radiotherapy (20 Gy) was delivered on days 3, 6, 9, and 12 to simulate a clinical hypofractionated radiotherapy schedule.

**Figure 2:**
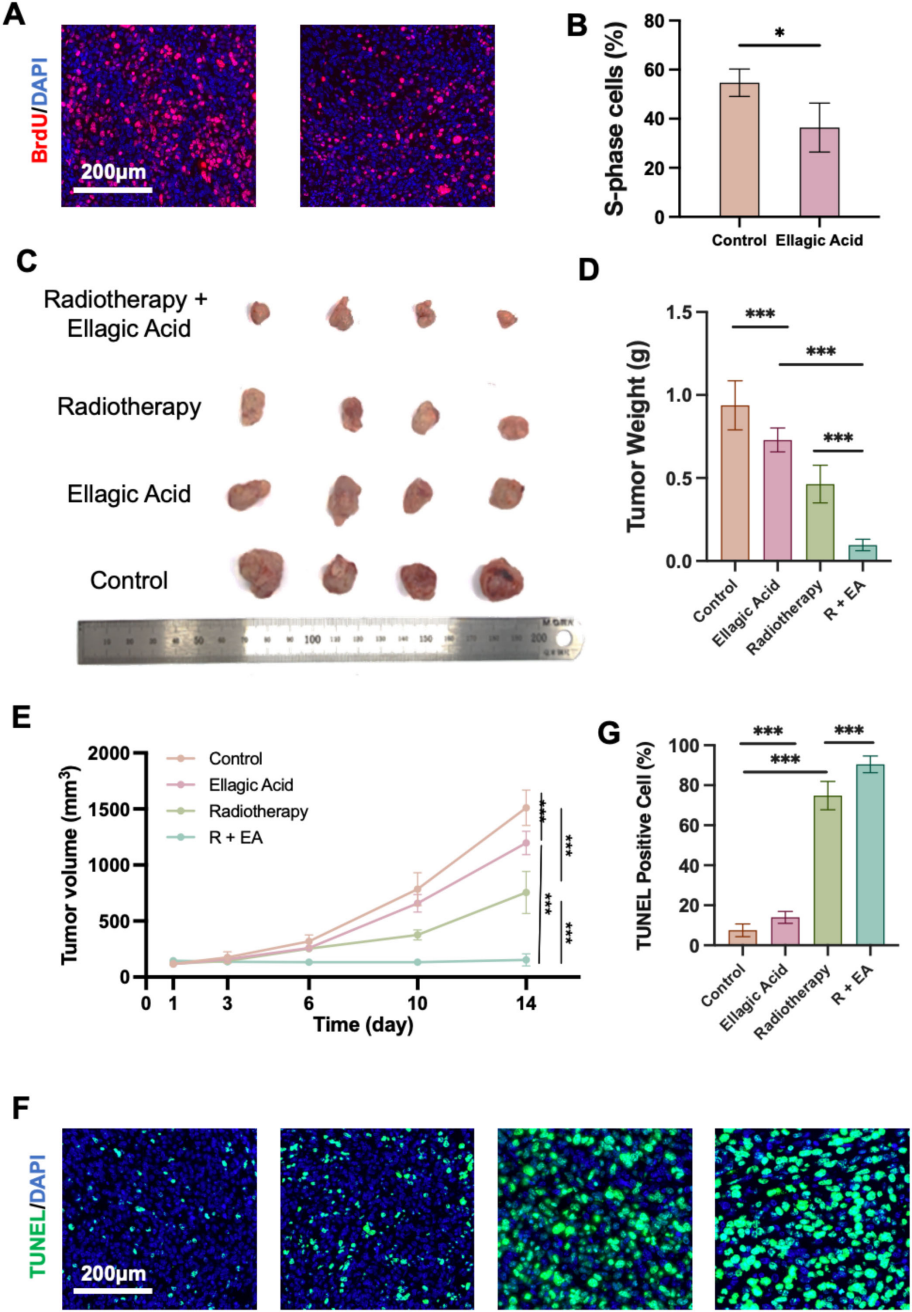
Evaluation of ellagic acid–mediated radiosensitization in a 4T1 TNBC mouse model. **(A)** BrdU/DAPI immunofluorescence staining of tumor sections on day 3. **(B)** Quantification of S-phase cells based on BrdU incorporation. **(C)** Gross morphology of tumors collected at endpoint (day 14). **(D)** Tumor weight at endpoint. **(E)** Tumor growth curves over a 14-day observation period. **(F)** TUNEL/DAPI staining of tumor sections on day 14. **(G)** Quantification of TUNEL-positive apoptotic cells. *P < 0.05, ***P < 0.001; ns, not significant. n = 5 mice per group.

To determine whether ellagic acid suppresses cell cycle progression *in vivo*, a subset of tumors was harvested on day 3 for BrdU incorporation analysis. BrdU, a thymidine analog, incorporates into newly synthesized DNA during the S phase and serves as a marker of DNA replication. Immunofluorescence staining revealed a significantly lower proportion of BrdU-positive S-phase cells in the ellagic acid group compared to controls (**Figures 2A, B**), indicating *in vivo* G1 arrest and suppression of DNA synthesis.

Macroscopic tumor comparisons at the experimental endpoint are shown in **Figure 2C**. Quantitative analysis revealed that the combination of ellagic acid and radiotherapy (R+EA) led to the lowest tumor weight among all groups (**Figure 2D**). Tumor growth curves (**Figure 2E**) further demonstrated that the R+EA group exhibited the most potent tumor growth suppression across the observation period.

Given that DNA double-strand breaks are the primary mechanism of radiotherapy-induced cellular injury (22), we evaluated apoptosis in tumor tissues via TUNEL staining on day 14. TUNEL specifically labels DNA fragmentation, a hallmark of apoptosis (26). Immunofluorescence imaging (**Figure 2F**) and quantitative analysis (**Figure 2G**) revealed a significant increase in TUNEL-positive cells in the R+EA group, suggesting enhanced radiation-induced apoptosis.

Collectively, these results demonstrate that ellagic acid enhances radiosensitivity *in vivo* by promoting G1 arrest and amplifying radiotherapy-induced apoptotic responses, thereby significantly inhibiting tumor progression. These findings support the therapeutic potential of ellagic acid as a radiosensitizing agent in cancer treatment.

### 2.3 Ellagic acid attenuates radiotherapy-induced myocardial injury and preserves cardiac function

Although radiotherapy remains a cornerstone treatment for TNBC, its clinical application is often complicated by off-target effects, including RIMI, especially during thoracic irradiation (5). Given the increasing prevalence of TNBC patients receiving chest-directed radiotherapy, mitigating cardiotoxicity has become a critical therapeutic concern. To evaluate the cardioprotective potential of ellagic acid under these conditions, we performed histological and functional assessments in a TNBC-bearing mouse model subjected to combined treatment.

**Figure 3:**
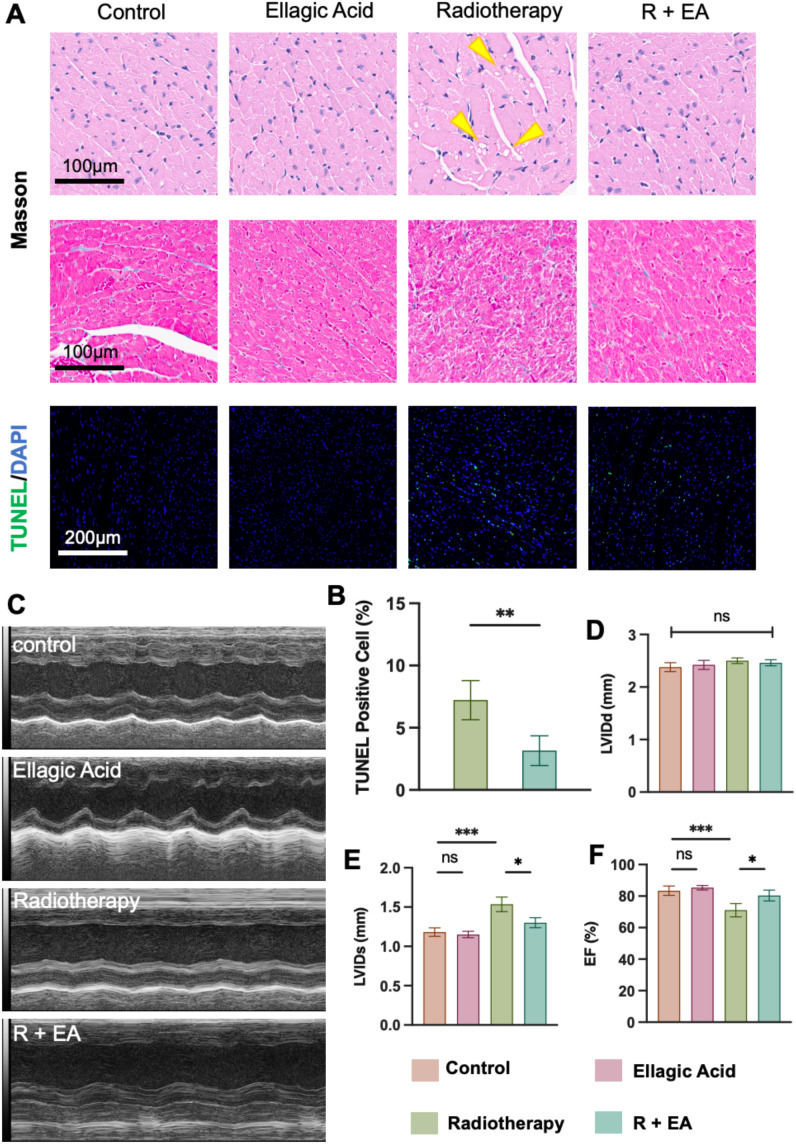
Histological and functional assessment of radiation-induced myocardial injury and the cardioprotective effect of ellagic acid. **(A)** Representative images of cardiac tissue stained with H&E, Masson, and TUNEL/DAPI. Radiotherapy caused vacuolization (yellow arrows), structural disarray, and increased apoptosis, while ellagic acid co-treatment alleviated these changes. **(B)** Quantification of TUNEL-positive cardiomyocytes. **(C)** M-mode echocardiography images of left ventricular motion. **(D)** Left ventricular internal diameter in diastole (LVIDd). **(E)** Left ventricular internal diameter in systole (LVIDs). **(F)** Left ventricular ejection fraction (EF%). *P < 0.05, **P < 0.01, ***P < 0.001; ns, not significant. n = 5 mice per group.

At the end of the experiment, heart tissues were collected and subjected to hematoxylin–eosin (HE), Masson trichrome, and TUNEL staining (**Figure 3A**). HE staining showed evident vacuolization in cardiomyocytes (yellow arrows) in the radiotherapy group. Masson staining revealed disorganized myocardial fiber alignment after irradiation. Notably, the combination group (R+EA) showed clear alleviation of these morphological abnormalities. Moreover, the ellagic acid–only group exhibited no obvious histological alterations compared to controls, suggesting favorable cardiac safety.

TUNEL staining further revealed a marked increase in apoptotic cardiomyocytes in the radiotherapy group, which was significantly reduced in the R+EA group. Quantification confirmed this reduction in TUNEL-positive cells (**Figure 3B**).

To assess cardiac function, M-mode echocardiography was performed (**Figure 3C**). No significant differences were observed in the left ventricular internal diameter in diastole (LVIDd) across groups (**Figure 3D**). However, the left ventricular internal diameter in systole (LVIDs) was significantly increased in the radiotherapy group, indicating impaired contractility, while ellagic acid co-treatment reversed this change (**Figure 3E**). Correspondingly, the ejection fraction (EF%) was significantly decreased by radiotherapy but restored in the R+EA group (**Figure 3F**). The ellagic acid only group exhibited echocardiographic parameters comparable to the control group, further supporting the cardiac safety profile of ellagic acid.

Collectively, these results demonstrate that ellagic acid effectively mitigates radiation-induced structural myocardial damage, reduces cardiomyocyte apoptosis, and preserves left ventricular systolic function. Importantly, ellagic acid alone did not induce detectable cardiotoxicity, supporting its potential as a safe cardioprotective agent during radiotherapy.

### 2.4 Ellagic acid exhibits favorable safety profile and alleviates radiotherapy-induced systemic toxicity

To systematically evaluate the biosafety of ellagic acid and its influence on radiotherapy-induced systemic toxicity, we performed histopathological and serological assessments in major non-cardiac organs.

**Figure 4:**
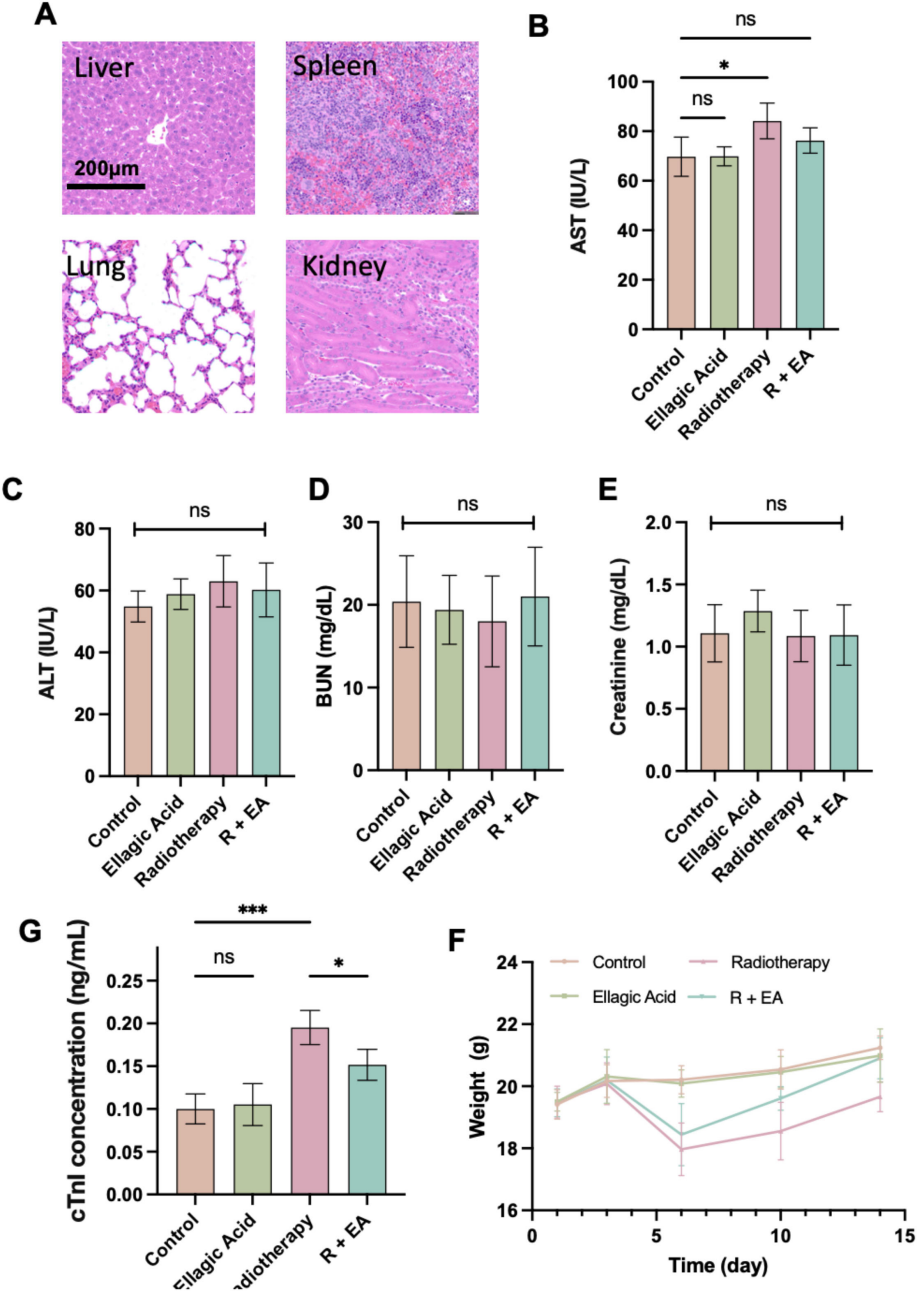
Evaluation of the systemic safety and toxicity profile of ellagic acid and its modulatory effect on radiotherapy-induced organ injury. **(A)** Representative H&E-stained sections of liver, spleen, lung, and kidney from each treatment group. **(B–E)** Serum levels of AST **(B)**, ALT **(C)**, BUN **(D)**, and creatinine **(E)**. **(F)** Body weight monitoring during treatment. **(G)** Serum cardiac troponin I (cTnI) concentrations. *P < 0.05, ***P < 0.001; ns, not significant. n = 5 mice per group.

Histological analysis of the liver, spleen, lung, and kidney via HE staining (**Figure 4A**) revealed no observable morphological abnormalities in the ellagic acid–treated group compared to the control group, indicating that ellagic acid did not induce structural toxicity in these major organs. Given that myocardial injury was already evaluated in **Figure 3**, these findings further support the systemic safety of ellagic acid under the tested regimen.

In serum biochemical tests, radiotherapy caused a mild but statistically significant increase in aspartate transaminase (AST), suggesting a degree of hepatic or cardiac enzyme leakage (**Figure 4B**). However, alanine transaminase (ALT), blood urea nitrogen (BUN), and creatinine levels showed no significant differences among the groups (**Figures 4C–E**). Importantly, ellagic acid alone had no measurable impact on any of these parameters, again confirming its favorable safety profile.

To further evaluate cardiac injury at the molecular level, we measured serum cardiac troponin I (cTnI), a more specific and sensitive marker for myocardial damage. Radiotherapy significantly elevated cTnI levels, while ellagic acid co-treatment (R+EA) effectively reduced this elevation (**Figure 4G**), suggesting that ellagic acid alleviates radiation-induced myocardial injury *in vivo*.

Finally, body weight was monitored throughout the treatment period. As shown in **Figure 4F**, radiotherapy led to a notable reduction in body weight during the early phase, reflecting systemic stress or toxicity. In contrast, ellagic acid co-administration ameliorated this decline, and mice in the R+EA group regained weight more rapidly. Notably, ellagic acid alone did not affect body weight, further supporting its metabolic safety.

Together, these data confirm that ellagic acid is a well-tolerated agent without inducing organ toxicity or affecting systemic physiology. Furthermore, it partially mitigates radiotherapy-associated hepatic enzyme elevation, weight loss, and myocardial injury, reinforcing its suitability as a radiotherapy adjuvant.

## 3 Discussion

This study is the first to demonstrate that ellagic acid, a naturally occurring polyphenol, exerts dual modulatory effects during radiotherapy: it enhances tumor radiosensitivity in TNBC while simultaneously protecting the myocardium from RIMI. This unique therapeutic profile, driven by tissue-specific mechanisms, addresses two critical challenges in TNBC radiotherapy—tumor radioresistance and treatment-associated cardiotoxicity—and holds significant translational potential.

Mechanistically, ellagic acid enhances the radiosensitivity of TNBC cells by inducing G1-phase arrest and impeding cell cycle progression (15–17). Our results show that ellagic acid markedly downregulates CDK4 expression and increases the proportion of cells in G1 phase, thereby limiting progression into the S phase—a cell cycle stage associated with enhanced DNA repair capacity and radioresistance. This finding aligns with current strategies that utilize CDK4/6 inhibitors to potentiate radiation efficacy by impairing DNA damage recovery (27). Importantly, ellagic acid achieves this effect via a distinct epigenetic mechanism: by inhibiting CARM1-mediated histone H3R17 dimethylation, ellagic acid disrupts the transcriptional activation of CDK4 at an upstream level (15–17). This mode of action not only deepens its regulatory influence over the cell cycle but also suggests broader chromatin-modifying capabilities that may benefit oncologic therapy.

Our *in vivo* data further support this mechanism. ellagic acid -treated tumors exhibited a significant reduction in S-phase cell populations and an increase in radiation-induced apoptosis, resulting in robust tumor growth inhibition. Concurrently, ellagic acid exhibited notable cardioprotective effects. Histopathological analyses revealed that ellagic acid alleviated radiation-induced myocardial vacuolization and structural disorganization, reduced the number of TUNEL-positive apoptotic cardiomyocytes, and preserved key functional parameters such as LVIDs and ejection fraction. Mechanistically, these protective effects are underpinned by ellagic acid’s potent antioxidant activity, as demonstrated by its free radical scavenging performance in chemical (e.g., DPPH) and cellular oxidative stress assays. Given that radiation-induced cardiac injury is primarily mediated by oxidative stress in non-proliferating cardiomyocytes, ellagic acid’s redox-modulating properties provide a biologically sound and clinically relevant basis for myocardial protection.

Remarkably, ellagic acid circumvents the conventional trade-off between radiosensitization and toxicity. Most radiosensitizers increase the risk of collateral damage to normal tissues, while radioprotectors may compromise tumor control (28, 29). Ellagic acid uniquely resolves this dilemma via cell-type–specific mechanisms: in tumor cells, it promotes radiosensitization by disrupting cell cycle progression; in cardiomyocytes, it mitigates oxidative injury without interfering with cell division (as cardiomyocytes are non-proliferative). This differential modulation represents a conceptual advance in the development of radiotherapy adjuvants and positions ellagic acid as a paradigm-shifting dual-function compound. Traditional agents like amifostine are designed solely to shield normal tissues from radiation damage. While effective, their use is often limited by systemic side effects (e.g., hypotension, nausea) and a theoretical concern of potentially protecting tumor cells, which could compromise radiotherapy efficacy (30, 31). In stark contrast, EA operates with tissue selectivity. It does not act as a blanket protector; instead, it exploits the fundamental biological difference between proliferating cancer cells and cardiomyocytes. This mechanistic dichotomy effectively decouples protection from tumor compromise, a major advancement over non-selective protectors.

The promising dual effects of EA demonstrated in our preclinical model necessitate a critical evaluation of its potential administration routes for future clinical application in TNBC radiotherapy. While intraperitoneal injection was effectively employed in our murine studies to establish proof-of-concept, translating this finding to the clinic requires strategic adaptation to meet practical, safety, and efficacy standards. The intraperitoneal route, though ensuring high bioavailability in rodents, is invasive and impractical for the repeated, long-term administration required during a typical radiotherapy course for human patients. Therefore, identifying a feasible and optimized clinical delivery method is paramount.

Oral administration stands as the most desirable and patient-compliant route. EA’s natural occurrence in certain foods suggests a favorable safety profile for oral intake (31–34). However, its notoriously poor aqueous solubility and moderate oral bioavailability present significant translational hurdles. To overcome this, future efforts must prioritize the development of advanced pharmaceutical formulations. Strategies such as nanocrystal technology, phospholipid complexes, cyclodextrin inclusion complexes, or nano-delivery systems (e.g., liposomes, polymeric micelles) could dramatically enhance EA’s solubility, stability, and intestinal absorption, thereby improving its systemic availability.

## 4 Conclusion

In summary, ellagic acid achieves a rare therapeutic duality: it sensitizes TNBC tumors to radiation by epigenetically suppressing CDK4-driven cell cycle progression, while protecting the heart via antioxidant-mediated mitigation of oxidative stress. This paradigm reconciles the competing goals of enhanced oncologic efficacy and reduced cardiotoxicity, offering a promising strategy to improve the therapeutic index of TNBC radiotherapy. Given its natural origin, safety, and multimodal activity, ellagic acid warrants rapid translation into clinical trials.

## 5 Materials and methods

Cell Culture and Reagents: The 4T1 breast cancer cells were obtained from Procell (Wuhan) and cultured in DMEM supplemented with 10% fetal bovine serum and 1% penicillin-streptomycin at 37 °C with 5% CO_2_. H9C2 cardiomyocytes were cultured under similar conditions. Ellagic acid was purchased from Yuanye (Shanghai) and dissolved in DMSO for *in vitro* experiments and in 0.5% CMC-Na for *in vivo* administration.

*In Vivo* Tumor Model and Treatment: Female BALB/c mice (8 weeks old, approximately 20 g) were subcutaneously inoculated with 1 × 10^6^ 4T1 cells into the left mammary fat pad, located in close proximity to the cardiac region of the thoracic wall. Once tumors reached approximately 0.1 cm³ in volume, this time point was designated as Day 0. From Day 1 onwards, mice in the ellagic acid treatment group received daily intraperitoneal injections of ellagic acid at a dose of 50 mg/kg for 14 consecutive days. For radiotherapy, mice were anesthetized and positioned supine under a linear accelerator. A single 20 Gy dose of 6 MV X-rays was delivered on Day 3, 6, 9,12 using a single anterior field, with the irradiation field encompassing both the tumor and the underlying cardiac region.

BrdU Incorporation Assay: To evaluate S-phase distribution *in vivo*, mice were injected intraperitoneally with BrdU (50 mg/kg) 2 hours prior to sacrifice. Tumors were harvested, fixed, embedded, and sectioned. BrdU incorporation was detected by immunofluorescence using anti-BrdU antibody (Abcam) and quantified with ImageJ software.

TUNEL Assay: Apoptotic cells in tumor and heart tissues were detected using the TUNEL apoptosis assay kit (Solarbio) according to manufacturer instructions. Nuclei were counterstained with DAPI. TUNEL-positive cells were quantified in at least five random fields per section.

Histological and Masson’s Trichrome Staining: Heart tissues were harvested on day 14, fixed in 4% paraformaldehyde, embedded in paraffin, and sectioned. HE staining was performed to evaluate myocardial architecture, and Masson’s trichrome staining was used to assess fibrosis. Histological alterations were evaluated under light microscopy by blinded pathologists.

Cardiac Function Assessment: Cardiac function was evaluated using transthoracic echocardiography (Vevo2100, VisualSonics) under light anesthesia. M-mode images were obtained at the level of the papillary muscles to determine LVIDd, LVIDs, and EF%. End Volume = 7 × LVID³/(2.4 + LVID), and EF (%) = (End-Diastolic Volume - End-Systolic Volume)/End-Diastolic Volume.

Western Blotting: Cells were lysed in RIPA buffer with protease/phosphatase inhibitors. Equal protein amounts were resolved by SDS-PAGE and transferred to PVDF membranes. Membranes were incubated with primary antibodies against CDK4, followed by HRP-conjugated secondary antibodies and chemiluminescent detection.

Cell Cycle Analysis: Cell cycle distribution was assessed by propidium iodide (PI) staining followed by flow cytometry. Cells were harvested at approximately 70–80% confluence, trypsinized, and washed twice with cold phosphate-buffered saline (PBS). The cell pellets were then fixed by dropwise addition of prechilled 70% ethanol while gently vortexing and stored at –20 °C for at least 2 hours. After fixation, cells were washed once with PBS to remove residual ethanol and incubated in a staining solution containing 50 µg/mL propidium iodide (PI) and 100 µg/mL RNase A in PBS for 30 minutes at 37 °C in the dark. PI intercalates into double-stranded DNA, and the fluorescence intensity correlates directly with DNA content: G0/G1 phase cells exhibit 2N DNA content, S-phase cells show intermediate DNA content (between 2N and 4N due to active replication), and G2/M phase cells have 4N DNA content.

DPPH Free Radical Scavenging Assay: The antioxidant activity of ellagic acid was evaluated using the DPPH assay. Ellagic acid solutions of various concentrations (1–50 µM) were incubated with 0.2 mM DPPH in methanol at room temperature in the dark. Absorbance at 517 nm was measured after 30 minutes. Scavenging activity was calculated as a percentage of control.

Cell Viability and Clonogenic Survival Assay: Cells were seeded into 96-well plates at a density of 5 × 10³ cells/well and allowed to adhere overnight. Cells were then treated with ellagic acid (various concentrations) for 24 hours prior to irradiation, followed by exposure to 6 Gy X-ray. After irradiation, the culture medium was replaced with fresh medium without ellagic acid, and the cells were incubated for an additional 24 hours. Cells viability was assessed using the Cell Counting Kit-8 according to the manufacturer’s instructions. Absorbance was measured at 450 nm using a microplate reader.

Blood Biochemistry: Serum samples were collected from orbital blood on day 14. AST, ALT were measured using an automatic biochemical analyzer. cTnI levels were measured using a commercially available ELISA kit according to the manufacturer’s instructions.

Statistical Analysis: All data are presented as mean ± SEM. Statistical significance was evaluated using Student’s t-test or one-way ANOVA followed by Tukey’s *post-hoc* test. A p-value < 0.05 was considered statistically significant.

## Data Availability

The raw data supporting the conclusions of this article will be made available by the authors, without undue reservation.
