# Supplementary material for: A radiotherapeutic paradox: ellagic acid sensitizes tumors while attenuating radiation-induced myocardial injury
Source: Front Oncol. 2026 Jan 28;16:1652278. doi: 10.3389/fonc.2026.1652278 (PMC12890680; doi:10.3389/fonc.2026.1652278)
Supplement: Supplementary file 1 [file DataSheet1.docx]

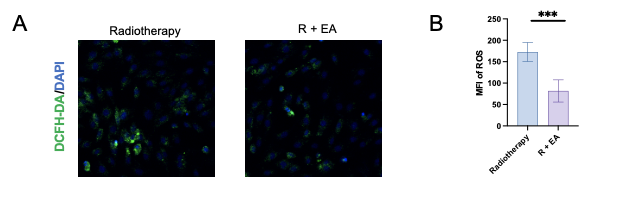


**Figure S1. Ellagic acid mitigates irradiation-induced oxidative stress in H9C2 cardiomyocytes.** **(A)** Representative fluorescent images showing intracellular ROS levels in H9C2 cells. Cells were treated as follows: (i) exposed to Co60 γ-irradiation (IR) alone, and (ii) exposed to IR followed immediately by EA treatment. Cells were stained with a ROS-sensitive fluorescent dye 2 hours post-irradiation. **(B)** Quantitative analysis of the mean fluorescence intensity (MFI) of ROS from panel (A). Data are presented as mean ± SD (n = 3). ***p < 0.001 (statistical significance determined by one-way ANOVA with Tukey's post hoc test).
